# Conflict over Male Parentage in Social Insects

**DOI:** 10.1371/journal.pbio.0020248

**Published:** 2004-08-24

**Authors:** Robert L Hammond, Laurent Keller

**Affiliations:** **1**Department of Ecology and Evolution, Bâtiment de BiologieUniversity of Lausanne, LausanneSwitzerland

## Abstract

Mutual policing is an important mechanism that maintains social harmony in group-living organisms by suppressing the selfish behavior of individuals. In social insects, workers police one another (worker-policing) by preventing individual workers from laying eggs that would otherwise develop into males. Within the framework of Hamilton's rule there are two explanations for worker-policing behavior. First, if worker reproduction is cost-free, worker-policing should occur only where workers are more closely related to queen- than to worker-produced male eggs (relatedness hypothesis). Second, if there are substantial costs to unchecked worker reproduction, worker-policing may occur to counteract these costs and increase colony efficiency (efficiency hypothesis). The first explanation predicts that patterns of the parentage of males (male parentage) are associated with relatedness, whereas the latter does not. We have investigated how male parentage varies with colony kin structure and colony size in 50 species of ants, bees, and wasps in a phylogenetically controlled comparative analysis. Our survey revealed that queens produced the majority of males in most of the species and that workers produced more than half of the males in less than 10% of species. Moreover, we show that male parentage does not vary with relatedness as predicted by the relatedness hypothesis. This indicates that intra- and interspecific variation in male parentage cannot be accounted for by the relatedness hypothesis alone and that increased colony efficiency is an important factor responsible for the evolution of worker-policing. Our study reveals greater harmony and more complex regulation of reproduction in social insect colonies than that expected from simple theoretical expectations based on relatedness only.

## Abstract 2

Workers of social insects prevent other workers laying eggs to increase colony efficiency and not -- as traditionally thought - purely because workers are more related to the queen of the colony

## **Introduction**

Major evolutionary transitions (Maynard-Smith and Szathmáry 1995) require the evolution of mechanisms that moderate within-group conflict (Keller 1999; Queller 2000; Michod and Roze 2001). One such mechanism is mutual policing, where members of a group collectively prevent individuals from acting in their own selfish interests (Frank 1995). The best example of mutual policing behavior in nature is found in social insects, where workers police worker reproduction (worker-policing) by selectively removing worker-laid eggs that would otherwise develop into males (Ratnieks and Visscher 1989; Foster and Ratnieks 2000, 2001a; Halling et al. 2001; Oldroyd et al. 2001), or by directing aggression toward workers with developing ovaries (Monnin and Ratnieks 2001; Iwanishi et al. 2003). Selection for worker-policing depends upon two variables: the relative relatedness of workers to queen- and worker-produced males (relatedness hypothesis) and the colony-level cost of workers reproducing (efficiency hypothesis). Worker-policing theory (Starr 1984; Woyciechowski and Lomnicki 1987; Ratnieks 1988), an extension of kin selection theory (Hamilton 1964), has typically highlighted relatedness as the all-important variable that explains when workers should lay male-destined eggs and when they should police one another's reproduction. In contrast, the costs of worker reproduction (Ratnieks 1988) have been largely ignored or given low prominence in the literature, with the effect that the relatedness hypothesis has become widely accepted as the explanation for worker-policing (Whitfield 2002).

Empirical investigations of worker-policing behavior initially focused on species with colony kin structures that predicted the behavior under the relatedness hypothesis, and worker-policing was first demonstrated in the multiply mated honey bee, Apis mellifera (Estoup et al. 1994; Visscher 1996). Subsequently, similar patterns have been found in other multiply mated members of the genus *Apis* (Halling et al. 2001; Oldroyd et al. 2001; Wattanachaiyingcharoen et al. 2002) and in the multiply mated wasp Vespula vulgaris (Foster and Ratnieks 2001a). Support for the relatedness hypothesis comes from contrasts between these species and closely related species that are singly mated (Peters et al. 1999; Foster and Ratnieks 2001c) and from an intraspecific study of the vespine wasp *Dolichovespula saxonica,* in which worker-policing behavior is facultative and occurs only in colonies headed by multiply mated queens (Foster and Ratnieks 2000). There are, however, problems with the conclusion that relatedness is the underlying cause of policing behavior, because phylogeny is not controlled for in the interspecific comparisons described above. This is an important problem, because these species are clustered with respect to phylogeny (e.g., four *Apis* species), and related wasp species, such as *Vespa crabro,* show patterns of worker reproduction and worker-policing behavior that are consistent with the efficiency hypothesis but not the relatedness hypothesis (Foster et al. 2002).

The relatedness hypothesis explicitly predicts that the parentage of males (male parentage) is dependent upon colony kin structure. Importantly, males should be worker-produced in colonies headed by single, once-mated queens, and queen-produced in colonies headed by multiple related queens, or by multiply mated queens, because worker reproduction is prevented by worker-policing. By contrast, the efficiency hypothesis predicts no association of male parentage or worker-policing with colony kin structure. In this paper we test these predictions by analyzing, using methods that control for phylogenetic dependence, how the proportion of worker-produced males (WPM) varies with both colony kin structure and colony size. The theoretical difference in relatedness of workers to queen- and worker-produced males (*r*_diff_) was used to make predictions about male parentage based upon colony kin structure. We included colony size in our analyses because it potentially alters expected patterns of male parentage (Bourke 1999) by altering power relationships within the colony. In small colonies a single individual may have the power to dominate male production completely, but such reproductive dominance becomes less likely as colony size increases.

## **Results**

**Figure 1:**
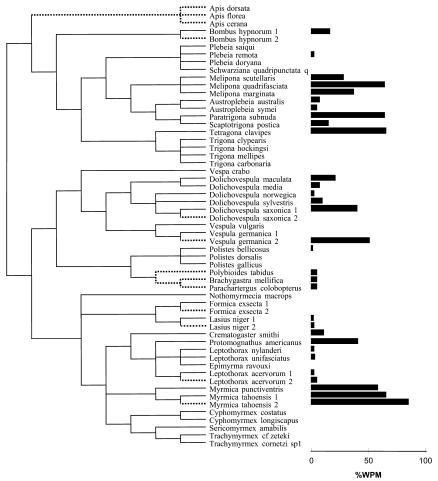
Composite Phylogeny Used in Comparative Analyses Phylogeny includes within-species variation. Duplicated species labeled 1 or 2 (e.g., Leptothorax acervorum 1 and 2) refer to taxa in which within-species variation was included in some analyses (see text for details). Dotted lines, *r*_diff_ is negative; solid lines, *r*_diff_ is positive. Horizontal bars indicate WPM.

We found data for 50 species: 16 ants, 20 bees, and 14 wasps (Table 1; Figure 1). WPM varied considerably (0%–85%), but in most species, queens produced the majority of males, with less than 10% of males being worker-produced in 72% of species surveyed. In only 10% of species were more than 50% of males worker-produced. There was great variation in the number of males (*n*_m_ = 13–1,426) and likewise in the number of assignable males (*n*_a_ = 10–677, where *n*_a_ is the sample size corrected for the probability of nondetection [Foster et al. 2001]) that were used to estimate the WPM. However, in those species for which we had relevant data, there was no significant correlation of *n*_m_ or *n*_a_ with WPM (Spearman's rank correlation: *n*_m_ versus WPM: *ρ* = 0.17, n = 45, *p* = 0.27; *n*_a_ versus WPM: *ρ* = 0.11, n = 27, *p* = 0.59), suggesting that there was no systematic bias in our dataset.

**Table 1:**
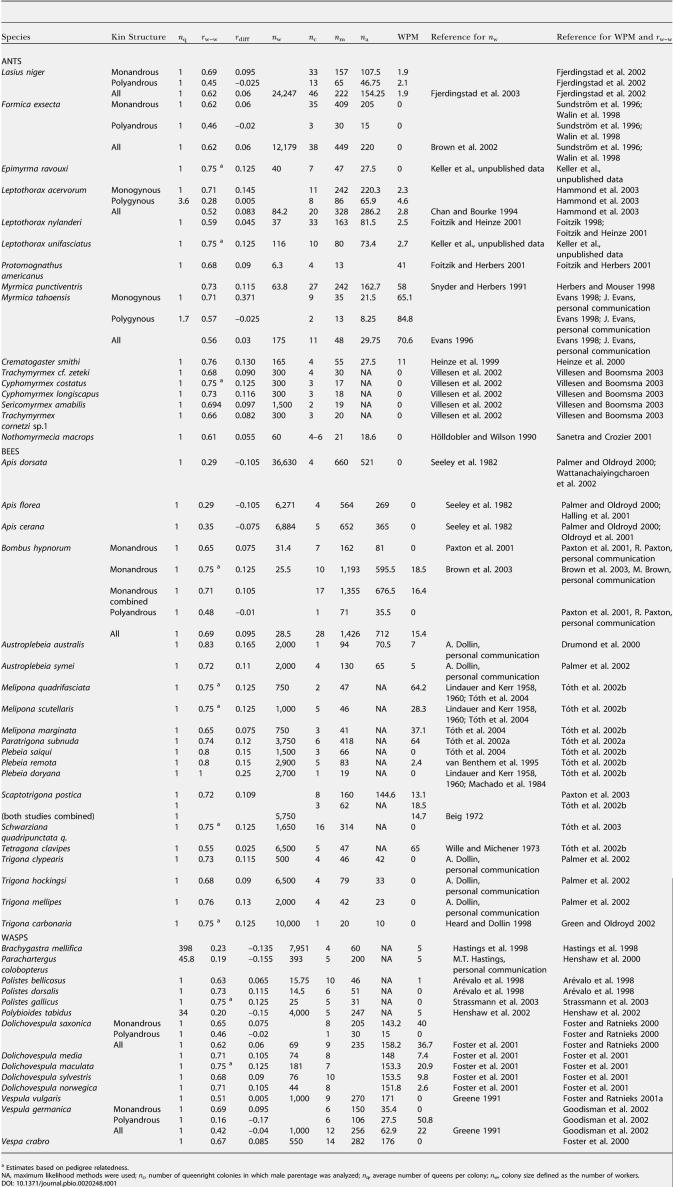
The WPM, Colony Kin Structure, and Colony Size in a Sample of Queenright Colonies of Eusocial Hymenoptera

^a^ Estimates based on pedigree relatedness

NA, maximum likelihood methods were used; *n*_c_, number of queenright colonies in which male parentage was analyzed; *n*_q_, average number of queens per colony; *n*_w_, colony size defined as the number of workers

### Comparative Analysis

Tests of serial independence showed that there was significant phylogenetic dependence for all variables when within-species variation was ignored (log_10_WPM, *p* = 0.016; *r*_diff_, *p* < 0.001; log_10_ of colony size [log_10_ *n*_w_], *p* < 0.001) and when within-species variation was included (log_10_WPM, *p* = 0.002; *r*_diff_, *p* < 0.001). This confirmed that a comparative approach using an analysis of independent contrasts was warranted (Abouheif 1999; Freckleton et al. 2002).

**Figure 2:**
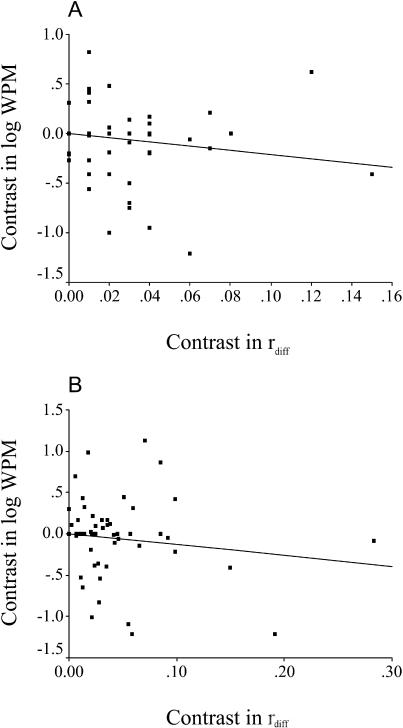
Variation in Worker Reproduction with Colony Kin Structure Axes show standardized independent contrasts in WPM (log_10_WPM) and in *r*_diff_. (A) is based on species values; (B) includes intraspecific variation for seven species (see text). Lines of regression are forced through the origin.

**Figure 3:**
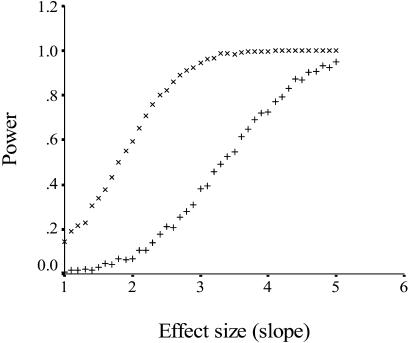
Statistical Power As a Function of the Slope *β* (Effect Size) in Comparative Analyses of *r*_diff_ on WPM On the graph, + data points show the power of tests in which within-species variation was ignored, and × show the power of tests in which within-species variation was included.

**Figure 4:**
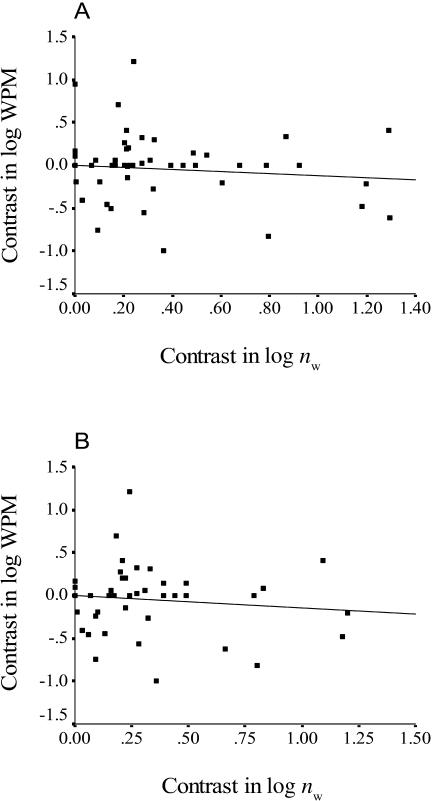
Variation in Worker Reproduction with Colony Size Axes show standardized independent contrasts in the proportion of worker-produced males (log_10_WPM) and in colony size (log_10_ *n*_w_). (A) includes all species; (B) includes only species in which relatedness predicts worker-produced males (i.e., *r*_diff_ is positive). Lines of regression are forced through the origin.

The WPM was not significantly correlated with colony kin structure in any of our comparative analyses. Ignoring within-species variation, the slope of the line of regression of contrast in log_10_WPM against contrast in *r*_diff_ was not significantly different from zero (Figure 2A; slope *β* = −2.14, *t* = –1.53, df = 48, *p* = 0.13), and the mean contrast in log_10_WPM (–1.70 ± 5.4) was not significantly different from zero when *r*_diff_ was coded categorically (*t* = 0.31, df = 2, *p* = 0.78). Likewise, neither analysis that included within-species variation was significant (Figure 2B; *β* = –1.31, *t* = –1.36, df = 55, *p* = 0.18; mean contrast in log_10_WPM = –0.14 ± 4.81, *t* = 0.03, df = 7, *p* = 0.98). The power was high (Figure 3; power greater than 0.75) for both analyses of regression to detect a large effect of relatedness on WPM, and there was relatively high power (see Figure 3; power greater than 0.6) to detect a medium effect in the analysis that included within-species variation. The WPM also did not show any significant relationship with colony size when all species were included (Figure 4A; *β* = –0.12, *t* = –1.04, df = 48, *p* = 0.30) or when relatedness was controlled for and we included only species with positive *r*_diff_ values (Figure 4B; *β* = –0.14, *t* = –1.05, df = 41, *p* = 0.30).

## **Discussion**

Our survey revealed that queens produced the majority of males in most of the species, and in less than 10% of the species did workers produce more than half of the males, in line with earlier surveys based largely on behavioral data (Bourke 1988; Choe 1988). Since workers of all the species included in our survey have functional ovaries, this demonstrates that self-restraint and worker-policing are widespread and powerful mechanisms that regulate reproduction in colonies of social Hymenoptera.

Our comparative study did not support the view that intra- and interspecific variation in male parentage can be accounted for by the relatedness hypothesis only. First, and most importantly, the proportion of males produced by workers was not significantly associated with colony kin structure. This was true both when within-species variation in colony kin structure was included and when it was ignored. In fact, although the relatedness hypothesis predicts a positive relationship between WPM and *r*_diff_, the analyses of relatedness revealed a tendency for a negative relationship. Importantly, our study included data from 50 species, and our power analyses showed that we had enough power to detect a relationship between male parentage and colony kin structure if it was of moderate or large effect.

A second line of evidence against the relatedness hypothesis came from the finding that workers produce only very few males in a large number of species where, on purely relatedness grounds, they would benefit from producing males. Workers produce less than 10% of males in 30 of the 43 species (70%) in which workers were more related to worker-produced than to queen-produced males.

A third line of evidence came from within-species comparisons. Only in Dolichovespula saxonica (Foster and Ratnieks 2000) were patterns of male parentage compatible with the relatedness hypothesis. By contrast, patterns of male parentage contradicted the relatedness hypothesis in the ants Leptothorax acervorum (Hammond et al. 2003), Lasius niger (Fjerdingstad et al. 2002), Formica exsecta (Sundström et al. 1996; Walin et al. 1998), and Myrmica tahoensis (Evans 1998). Interestingly, intraspecific variation in colony sex ratios in agreement with relatedness predictions have been shown in L. acervorum (Chan and Bourke 1994; Chan et al. 1999; Hammond et al. 2002), F. exsecta (Sundström et al. 1996), and M. tahoensis (Evans 1995, 1998). This suggests that although workers in these species can assess within-colony relatedness, they do not appear to respond to it in the context of the conflict over male parentage (Walin et al. 1998; Hammond et al. 2003).

The lack of association between kin structure and the degree of male parentage by workers indicates that factors others than relatedness effectively act as a brake on worker reproduction. The finding of no significant effect of colony size on WPM suggests that the ratio of queens to workers is not an important general factor regulating reproductive division of labor in social Hymenoptera. The low instance of worker reproduction is therefore unlikely to be the consequence of queens using aggression or pheromones to suppress worker reproduction, except, perhaps, in the few species with very small numbers of workers (e.g., Strassmann et al. 2003).

Most importantly, unchecked worker reproduction is likely to reduce overall colony productivity and may therefore reduce the average fitness of colony members. For example, reproductive workers have been found to spend time engaged in dominance interactions and egg-laying (Cole 1986) that otherwise would be used for foraging and brood rearing. Unchecked worker reproduction could also cause a “tragedy of the commons” (Hardin 1968; Frank 1995, 1996), because there would be more male brood than can be reared by the colony. If queens conceal the sex of their eggs (Nonacs 1993), these costs may also include workers mistakenly replacing queen-laid diploid eggs with their own male eggs. Furthermore, costs incurred by workers biasing colony sex ratios can select for worker-policing behavior (Foster and Ratnieks 2001b). Theory shows that these costs do not have to be large for worker-policing and self-restraint to be selected (Ratnieks 1988).

Our data showed considerable variation across species in the origin of males, raising the question, what are the factors underlying interspecific variation in male parentage? The efficiency hypothesis predicts that the extent of worker-produced males should depend largely on the shape and slope of the function relating colony productivity and worker efficiency. This property is expected to vary across species, and it is conceivable that closely related species, which are likely to live in similar habitats and have similar life histories, also have similar functions relating colony productivity and worker efficiency. Consistent with this prediction, our analysis revealed a significant phylogenetic signal, with closely related species being more similar in terms of the origin of males than expected by chance. Importantly, this similarity was not due to a greater similarity in kin structure and colony size between closely related species, because these two factors had no significant effect on the origin of males.

Previous evidence for the view that the relatedness hypothesis can account for variation in male parentage comes mostly from matched comparisons between honey bees (genus *Apis*) and singly mated stingless bees (tribe Meliponini) (Ratnieks 1988; Peters et al. 1999) and comparisons within vespine wasps (Foster and Ratnieks 2001c). However, a closer inspection of these matched comparisons reveals problems. In the matched comparison with honey bees, stingless bees are generally assumed to have worker-produced males. However, there is considerable variation in levels of worker reproduction, with males in the majority of species being exclusively queen-produced (Figure 1). Moreover, workers of some stingless bee species are completely sterile (Suka and Inoue 1993; Boleli et al. 2000), indicating that considering stingless bees as a taxon with generalized worker reproduction is not warranted. Similarly, the matched comparison in vespine wasps also has problems. It is true that males are queen-produced, and that workers police one another in *Vespula vulgaris,* a species in which queens are multiply mated (Foster and Ratnieks 2001a), whereas at least some males are worker-produced in *Dolichovespula,* a species in which queens are singly mated (Foster et al. 2001). However, the wasp most basal in the phylogeny *(Vespa crabro)* is singly mated, yet males are all queen-produced because workers police one another (Foster et al. 2000, 2002). Considering *Vespa, Vespula,* and *Dolichovespula* together, the most parsimonious explanation is that worker-policing is the ancestral state in vespines and it has been lost, or at least reduced, in *Dolichovespula.* In short, neither of these traditional lines of support for the relatedness hypothesis stand up to close scrutiny.

In conclusion, our comparative analysis does not support relatedness as the general explanation of patterns of male parentage and occurrence of worker-policing in social Hymenoptera. The concentration of published examples of worker-policing in multiply mated bees and wasps probably reflects the influence of the relatedness hypothesis on the selection of study taxa, rather than relatedness being the ultimate explanation of worker-policing. Moreover, recent studies have revealed worker-policing in species in which the relatedness hypothesis predicts males to be produced by workers (Kikuta and Tsuji 1999; Foster et al. 2002; Hartmann et al. 2003; Iwanishi et al. 2003). We conclude that costs associated with worker reproduction are likely to be significant and variation in these costs to be the main factor underlying differences across species in the origin of males. Experimental investigations of the colony-level costs of worker reproduction have begun (Lopez-Vaamonde et al. 2003). More are needed. It will also be important to conduct behavioral assays to determine whether worker-policing, by either egg-eating or aggression toward workers with developing ovaries, is responsible for the lack of worker reproduction in the stingless bee genera *Trigona* and *Plebeia.* Finally, we would like to stress that the finding that kin structure alone cannot account for the intra- and interspecific variation in male parentage does not amount to saying that kin structure is unimportant. Rather, it may work in concert with costs as a force influencing patterns of male parentage in social insects. Thus, this study reveals greater harmony and more complex regulation of reproduction in social insect colonies than that expected from simple theoretical expectations based on relatedness alone.

## Materials and Methods

### 

#### **Male parentage**

For all analyses, the response variable was WPM (see Table 1). For almost all studies, estimates of WPM took into account the power of the genetic markers to detect worker reproduction using either exclusion (Foster et al. 2001) or maximum likelihood approaches (Arévalo et al. 1998). Where this type of analysis was not included in the original paper we reanalyzed data using the exclusion-based approach of Foster et al. (2001). Specific details of how we treated data are given for each species in Protocol S1.

With comparative analyses there is always the difficult question of deciding “quality control” criteria to ensure that data are reliable and comparable. We collated data from published, in-press, and unpublished sources where colony genetic structure and male parentage were known accurately from molecular genetic markers. We restricted our survey to those including molecular genetic data, because recent genetic studies have shown that colony kin structures inferred from behavioral observations are often incorrect (e.g., mating frequency in Leptothorax nylanderi c.f. Plateaux 1981; Foitzik et al. 1997; Foster and Ratnieks 2001c), and in some social insect taxa (e.g., stingless bees and ants), workers lay trophic eggs that mistakenly could be counted as reproductive (Bourke 1988). We also restricted our analysis to queen-containing (queenright) colonies and species in which workers have ovaries. We did this because our aim was to investigate the outcome of worker–queen and worker–worker conflict. For those studies that included data on both queenright and queenless colonies, we considered male parentage in queenright colonies only (Protocol S1; e.g., Vespula germanica [Goodisman et al. 2002]). For all but two species, Leptothorax unifaciatus and Epimyrma ravouxi (L. Keller, J. Heinze, and A. F. G. Bourke, unpublished data), data were for adult or pupal males. For these two exceptional species, we had estimates of WPM at only the egg stage. However, as we found few worker-laid male eggs in both species (see Table 1), our estimate of WPM at the egg stage most likely reflected WPM in adults. In our comparative analyses we used log_10_WPM.

#### **Colony genetic structure**

We made predictions about the parentage of males based on colony kin structure by calculating *r*_diff_, the theoretical difference in relatedness of workers to queen- (*r*_w–qm_) and to worker-produced males (*r*_w–wm_) (see Table 1). The relatedness hypothesis predicts that if *r*_diff_ is positive, males are worker-produced, and if *r*_diff_ is negative, males are queen-produced, because workers should police one another. For colonies headed by single queens, where variation in colony genetic structure is caused by variation in the effective mating frequency of queens (Pamilo 1993), we calculated *r*_diff_ as (2*r*_w–w_ – 1)/4, where *r*_w–w_ is the relatedness among adult workers. For species with variation in queen number (polygyny), predictions about worker reproduction are more complicated because both queen number and queen relatedness are important (Pamilo 1991). For these species, we estimated *r*_diff_ from the actual relatedness of workers to queens (*r*_w–q_) and among workers (*r*_w–w_) as *r*_diff_ = (*r*_w–w_ – *r*_w–q_)/2. In our comparative analyses we used *r*_diff_ as a continuous explanatory variable, or we coded *r*_diff_ categorically as one when *r*_diff_ was greater than zero (worker-produced males predicted), or as zero when *r*_diff_ was less than zero (queen-produced males predicted).

#### **Colony size**

We defined colony size as the number of adult workers per nest (*n*_w_; see Table 1). Where only ranges of worker number were given, we took the midpoint value, and if more than one estimate was available, we combined data by calculating unweighted means. In our comparative analysis we used log_10_ *n*_w_ as an explanatory variable.

#### **Comparative analysis**

We constructed an ant, bee, and wasp phylogeny (see Figure 1) by combining published phylogenies. For ants, we based our phylogeny on Keller and Genoud's (see Figure 3 in Keller and Genoud [1997]), which we modified in light of a recent combined molecular and morphological phylogeny (Ward and Brady 2003); for bees, we based it on a combined DNA and morphological phylogeny (see Figure 5 in Cameron and Mardulyn [2001]), and for wasps, on a morphological and behavioral phylogeny (Smith et al. 2001). In addition, we added phylogenetic details for the Meliponini (stingless bees) following Velthuis (1997), and for leptothoracine ants, we used the molecular phylogeny of Baur et al. (1996). We placed bees basal to ants and wasps (see Figure 1) (Brothers and Carpenter 1993; Brothers 1999). We set all branch lengths equal, corresponding to a punctuational view of evolutionary change, and we considered ambiguous nodes to be unresolved. Using this tree, we tested the assumption of the phylogenetic independence of our three variables (log_10_WPM, *r*_diff_, and log_10_ *n*_w_) by a test for serial independence (Abouheif 1999) calculated by the program Phylogenetic Independence (Reeve and Abouheif 2003). For these analyses, we rotated nodes within our dataset 10,000 times and randomly shuffled our data 10,000 times to generate our null distribution. As all three variables showed significant phylogenetic nonindependence (see Results), we used Felsenstein's method of independent contrasts in our comparative analyses (Felsenstein 1985).

Analyses using *r*_diff_ coded categorically were carried out using the “Brunch” algorithm in CAIC (Purvis and Rambaut 1995), whereas analyses using *r*_diff_ and log_10_ *n*_w_ coded as continuous variables were analyzed using the program PDTREE (Garland et al. 1999; Garland and Ives 2000). We tested Brunch analyses for significance by comparing the mean independent contrast against zero using *t*-tests. We tested for the significance of contrasts generated by PDTREE by regression through the origin. We did not reduce the number of degrees of freedom (df), as has been suggested for phylogenies containing polytomies (Purvis and Garland 1993), because none of our analyses were significant without such adjustment. Power analyses (see below) were calculated using R (http://www.r-project.org/). All other statistical tests were performed using SPSS (version 11).

We tested the hypothesis that colony kin structure determines patterns of male parentage both when within-species variation in kin structure was ignored and when it was included. In our first set of two analyses, we used estimates of WPM and *r*_diff_ that were mean values for each species. We calculated independent contrasts between log_10_WPM and *r*_diff_, and with *r*_diff_ coded as a categorical variable. In our second set of two analyses, we included within-species variation in colony genetic structure that was present in seven species because of facultative variation in queen number or queen mating frequency (see Table 1). We did this by calculating *r*_diff_ per colony and grouping colonies into those where *r*_diff_ was positive (worker-production of males was predicted), and those where *r*_diff_ was negative (males were predicted to be queen-produced because of worker-policing). We then estimated WPM for each group. We modified the phylogeny by adding an additional bifurcation at the tips corresponding to these seven species (see Figure 1). Although it is not necessary to control for phylogeny when testing hypotheses within species, doing so enabled us to combine evidence from within- and among-species comparisons (Garland et al. 1992). Using our modified dataset, we calculated independent contrasts between log_10_WPM and *r*_diff_, and with *r*_diff_ coded as a categorical variable.

We tested the role of colony size in two ways. First, we ignored any effect of relatedness and simply compared contrasts in log_10_WPM with contrasts in log_10_ *n*_w_. Second, we controlled for relatedness by limiting our analysis to species in which workers were more related to worker- than to queen-produced males (i.e., *r*_diff_ was positive), and then compared contrasts in log_10_WPM with contrasts in log_10_ *n*_w_ in this subset of the data.

#### **Statistical power**

To investigate the power of our analysis, we first determined the expected relationship between WPM and *r*_diff_ in our dataset. To do that we set WPM to 0% when *r*_diff_ was less than zero, to 100% when *r*_diff_ was greater than zero, and to 50% when *r*_diff_ was equal to zero. An analysis of independent contrasts based on this hypothetical relationship gave a highly significant relationship between WPM and *r*_diff_ both when within-species variation was ignored (*β* = 5.48, *t* = 6.57, df = 48, *p* < 0.0001) and included (*β* = 6.59, *t* = 9.12, df = 55, *p* < 0.0001). On the basis of these slopes, we conducted a power analysis by assuming two types of effects. We considered *r*_diff_ to have a “large” effect on WPM when *β* was greater than 4.0, and a “moderate” effect when *β* was between 2.0 and 4.0. To test the power that our analysis had to detect a large and moderate effect, we used the model *y* = *βx* + “resampled residual of *y*,” where *x* is the observed standardized contrast in r_diff_ and “resampled residual of *y*” is the residual of *y* estimated by resampling the distribution of residuals from our observed regressions through the origin. From this model, we defined power as the proportion of regressions (forced through the origin) in 1,000 simulated datasets that were significant at α ≤ 0.05 for a given slope *β* (the effect size). We investigated how power varied with effect size by increasing *β* incrementally from 1 to 5 in steps of 0.1 (see Figure 3).

## Supporting Information

Protocol S1 Details of Data Selection Methods and Sources A detailed synopsis of how data used in this paper were selected from published and unpublished sources.
(86 KB DOC).

## Abbreviations

df: degrees of freedom
log_10_*n*_w_: log_10_ of colony size
*n*_a_: number of assignable males
*n*_m_: total number of males analyzed genetically
*r*_diff_: the theoretical difference in relatedness of workers to queen- and worker-produced males
*r*_w–w_: relatedness among adult workers
WPM: proportion of worker-produced males
