# Supplementary material for: Conflict over Male Parentage in Social Insects
Source: PLoS Biol. 2004 Aug 24;2(9):e248. doi: 10.1371/journal.pbio.0020248 (PMC514489; doi:10.1371/journal.pbio.0020248)
Supplement: Protocol S1 — A detailed synopsis of how data used in this paper were selected from published and unpublished sources. (86 KB DOC). [file pbio.0020248.sd001.doc]

**Supplementary data**

**Conflict over Male Parentage in Social Insects**

**Robert L. Hammond and Laurent Keller**

**Data Selection.** Below we provide details of how we selected data from published sources, reanalyzed published data and analyzed unpublished results. For species not mentioned here, but included in Table 1, we took estimates directly from published papers without any modification.

**Ants**

***Leptothorax acervorum*.** We only considered estimates of the proportion of worker-produced males in male adults and pupae (Hammon*d et a*l. 2003).

***Leptothorax nylanderi*** (Foitzik 1998; Foitzik and Heinze 2001). We corrected for non-detection following Foster *et al.* (2001).

***Protomognathus americanus*** (Foitzik and Herbers 2001)**.** We only considered data from queenright fragments in this polydomous species (Foitzik and Herbers 2001). We ignored males of unknown parentage and incorporated the 6.3% non-detection error into our estimate proportion of worker-produced males.

***Myrmica punctiventris*** (Herbers and Mouser 1998)***.*** We recalculated the proportion of worker produced males, taking into account the non-detection error (Foste*r et a*l. 2001), using data on the number of informative loci, the number of males carrying at least one paternal allele and the total number of males supplied by the corresponding author (pers. com. J. Herbers). There was considerable variation in the proportion of worker-produced between the two study plots (65% in plot I, 10% in plot II). We combined these data to give an overall value.

***Formica exsecta.*** (Sundströ*m et a*l. 1996; Wali*n et a*l. 1998). We split colonies into two groups; M_e_ > 2 (n = 3 colonies) and M_e_ < 2 (n = 35 colonies) (Wali*n et a*l. 1998). We could not calculate non-detection error as the raw genotype data were not available to us. However, in 29 of 38 colonies all workers were heterozygous, whereas all males shared one genotype (see Table 3 in Wali*n et a*l. 1998). This suggested that there was at least one informative locus in these colonies. Given this, we estimated that the number of assignable males (N_a_) was approximately the 0.5 x number of males genotyped. (We did not consider *Formica truncorum*, *Formica rufa* and *Myrmica ruginodis*, which were included in Walin *et al’s* (1998) study, because these species were only genotyped at allozyme loci with limited variability (average of 2.3 alleles / locus), therefore, the power to detect male parentage was low).

***Nothomyrmecia macrops*** (Sanetra and Crozier 2001). We calculated the number of assignable males (Foste*r et a*l. 2001) from information on the number of informative loci supplied by the first author of the original paper (pers. com. M. Sanetra).

***Crematogaster smithi*** (Heinz*e et a*l. 2000). Data in table 2 of Heinze *et al.* (2000) corrected for non-detection following Foster *et al.* (2001).

***Trachymyrmex cf. zeteki, Cyphomyrmex costatus, Cyphomyrmex longiscapus, Sericomyrmex amabilis, Trachymyrmex cornetzi sp.1*** (Villese*n et a*l. 2002). We only considered male parentage in queenright colonies (see Table 1 in Villese*n et a*l. 2002) and we omitted data from two species (*Apterostigma collare* and *Trachymyrmex cornetzi sp.2*) where maximum likelihood analyses revealed there to be little power to determine whether males were queen or worker-produced (see Figure 1B in Villese*n et a*l. 2002).

***Lasius niger*.** We only considered data collected from the Lausanne population in 1997 (n = 22 colonies) and 1998 (n = 24 colonies) (see Fjerdingsta*d et a*l. 2003). For these colonies the first author of the original paper (pers. com. E. Fjerdingstad) reanalyzed the data to incorporate the probability of non-detection following Foster *et al.* (2001). For each colony we calculated the effective mating frequency (M_e_) as M_e_ = 2/(4r_ww_ -1)(Pamilo 1993) and grouped colonies according to if M_e_ > 2 (n = 13 colonies, no worker reproduction predicted) and those where M_e_ < 2 (n = 33 colonies, worker reproduction predicted).

***Myrmica tahoensis*** (Evans 1998). Of the 23 colonies that produced males, only in 11 were both workers and queens genotyped so allowing us to make predictions of male parentage based on relatedness (pers. com. J.Evans). This reduced the number of males for which parentage could be determined from 87 to 48. For this subset of colonies we compared relatedness among workers and of workers to colony queens and calculated r_diff_ as described in the methods section of the paper. Worker-produced males were predicted in 9 colonies (r_diff_ was positive) and queen-produced males and worker policing were predicted in 2 colonies (r_diff_ was negative).

***Leptothorax unifasciatus*** (Keller, Heinze & Bourke unpublished data). We analyzed the parentage of 80 haploid eggs, from 10 queenright colonies, which had been genotyped with up to 4 microsatellite loci. The genotypes of queens and their mates were found either from direct typing of the queen or by inference from the genotypes of multiple diploid eggs. On average, 17.4 eggs were genotyped per colony. From these colonies, we identified male eggs as eggs that appeared homozygous at all typed loci. The number of informative loci varied from 2-4 loci per colony. We corrected the proportion of worker-produced males for non-detection error following Foster *et al.* (2001).

***Epimyrma ravouxi*** (Keller, Heinze & Bourke unpublished data). We analyzed the parentage of 47 haploid eggs, from 7 queenright colonies, which had been genotyped with up to 6 microsatellite loci. We identified male eggs as for *L. unifasciatus.* For the 7 colonies an average of 11.9 eggs were typed per colony, and the number of informative loci varied from 1-3 loci per colony. We corrected for non-detection error following Foster *et al.* (2001).

**Bees**

***Austroplebeia australis*** (Drumon*d et a*l. 2000). We only included data from one (C2) of the two colonies. The queen and male in the second colony (C1) shared alleles at both loci so there were no informative loci with which to infer the parentage of males. However, the distribution of genotypes in males in colony C1 matched more closely that for males being queen-produced than that for males being worker-produced (Table 1 in Drumon*d et a*l. 2000).

***Austroplebeia symei*** (Palme*r et a*l. 2002). We quote the proportion of worker-produced males taken over all 4 colonies (5%, see Table 1), rather than just for 1 colony (13%) as quoted in the text of Palmer *et al.*(2002).

***Trigona carbonaria*** (Green and Oldroyd 2002). Males were produced in two colonies (colony 1 & 2, Table 1 in Green and Oldroyd 2002) but we included data only from 1 colony (colony 1) where there was an informative locus (Tc4.287). Although there were no informative loci in the second colony (colony 2), the distribution of genotypes a second colony (colony 2) supported the finding that males were exclusively queen-produced (Green and Oldroyd 2002).

***Scaptotrigona postica*** (Tót*h et a*l. 2002; Paxto*n et a*l. 2003). For data from Tóth *et al.* (2002) we estimated the number of worker-produced males for each colony as (n_m_*(1-Q)), where n_m_ is the number of males genotyped in each colony and Q is the likelihood proportion of males attributable to queens (Likelihood Q estimate in Tót*h et a*l. 2002). We then combined these values with data from 8 colonies that produced haploid males in Paxton *et al.’s* (2003) study to give an overall estimate.

***Plebeia saiqui, Plebeia remota, Plebeia doryana, Melipona quadrifasciata, Melipona scutellaris Melipona marginata, Tetragona clavipes*** (Tót*h et a*l. 2002). We used the same method as for *Scaptotrigona postica*.

***Bombus hypnorum*** (Paxto*n et a*l. 2001; Brow*n et a*l. 2003). We took data from table 2 in Paxton *et al.* (2001) and divided colonies into those with M_e_ < 2 (n = 7) and with M_e_ > 2 (n = 1). Data from colonies with M_e_ < 2 were combined with the 10 colonies headed by singly-mated queens in Brown *et al.* (2003) to give an overall value. We estimated mean worker relatedness (R_ww_) by calculating the arithmetic mean of colony relatedness from Table 2 in Paxton *et al.* (2001) and in Brown *et al.* (2003) assuming that R_ww_ = 0.75 for each colony in the latter study.

**Wasps**

***Polybioides tabidus*** (Hensha*w et a*l. 2002), ***Brachygastra mellifica*** (Hasting*s et a*l. 1998), ***Parachartergus colobopterus*** (Hensha*w et a*l. 2000). For these three species likelihood analysis showed that almost all males were queen-produced, although there was a possibility of a small amount of worker reproduction. In view of this we set the proportion of worker-produced males to 5% for each species respectively.

***Vespula germanica*** (Goodisma*n et a*l. 2002). We only included male producing colonies (n=12) from Goodisman *et al.* (2002) that contained diploid brood and so were most likely queenright when adult males were sampled (c.f. Foste*r et a*l. 2001). For these colonies we reanalyzed worker genotypes using the program Matesoft (Moilane*n et a*l. 2003). This identified the number of patrilines in each colony and assigned individual workers to particular patrilines. We used this data to calculate the number of assignable males (N_a_) following Foster *et al.* (2001). For each colony we calculated the effective mating frequency (M_e_, see methods for *Lasius niger*) and colonies were grouped into those where M_e_ > 2 (n = 6 colonies) and where M_e_ < 2 (n = 6 colonies).

***Dolichovespula saxonica*** (Foster and Ratnieks 2000). Based on effective mating frequency (see methods for *Lasius niger*) we split colonies into M_e_ < 2 (n = 8 colonies) and M_e_ > 2 (n = 1 colony).

***Polistes gallicus*** (Strassman*n et a*l. 2003). We only considered colonies (n = 5) where the queen was collected (see Table 1 in Strassman*n et a*l. 2003) to ensure that male parentage reflected the outcome of queen-worker conflict. We present data for male pupae only.

**References**

Brown MJF, Schmid-Hempel R, Schmid-Hempel P (2003) Queen-controlled sex ratios and worker reproduction in the bumble bee *Bombus hypnorum,* as revealed by microsatellites. Mol Ecol 12: 1599-1605.

Drumond PM, Oldroyd BP, Osborne K (2000) Worker reproduction in *Austroplebeia australis* Friese (Hymenoptera, Apidae, Meliponini). Insect Soc 47: 333-336.

Evans JD (1998) Parentage and sex allocation in the facultatively polygynous ant *Myrmica tahoensis*. Behav Ecol Sociobiol 44: 35-42.

Fjerdingstad EJ, Gertsch PJ, Keller L (2003) The relationship between multiple mating by queens, within-colony genetic variability and fitness in the ant *Lasius niger*. J Evol Biol 16: 844-853.

Foitzik S (1998) Population structure and sex allocation in the ant *Leptothorax nylanderi*. PhD thesis. Würzburg: University of Würzburg.

Foitzik S, Heinze J (2001) Microgeographic genetic structure and intraspecific parasitism in the ant *Leptothorax nylanderi*. Ecol Entomol 26: 449-456.

Foitzik S, Herbers JM (2001) Colony structure of a slavemaking ant. I. Intracolony relatedness, worker reproduction, and polydomy. Evolution 55: 307-315.

Foster KR, Ratnieks FLW (2000) Facultative worker policing in a wasp. Nature 407: 692-693.

Foster KR, Ratnieks FLW, Gyllenstrand N, Thorén PA (2001) Colony kin structure and male production in *Dolichovespula* wasps. Mol Ecol 10: 1003-1010.

Goodisman MAD, Matthews RW, Crozier RH (2002) Mating and reproduction in the wasp *Vespula germanica*. Behav Ecol Sociobiol 51: 497-502.

Green CL, Oldroyd BP (2002) Queen mating frequency and maternity of males in the stingless bee *Trigona carbonaria* Smith. Insect Soc 49: 196-202.

Hammond RL, Bruford MW, Bourke AFG (2003) Male parentage does not vary with colony kin structure in a multiple-queen ant. J Evol Biol 16: 446-455.

Hastings MD, Queller DC, Eischen F, Strassmann JE (1998) Kin selection, relatedness, and worker control of reproduction in a large-colony epiponine wasp, *Brachygastra mellifica*. Behav Ecol 9: 573-581.

Heinze J, Strätz M, Pedersen JS, Haberl M (2000) Microsatellite analysis suggests occassional worker reproduction in the monogynous ant *Crematogaster smithi*. Insect Soc 47: 299-301.

Henshaw MT, Queller DC, Strassmann JE (2002) Control of male production in the swarm-founding wasp, *Polybioides tabidus*. J Evol Biol 15: 262-268.

Henshaw MT, Strassmann JE, Quach SQ, Queller DC (2000) Male production in *Parachartergus colopterus,* a neotropical, swarmfounding wasp. Ethol Ecol Evol 12: 161-174.

Herbers JM, Mouser RL (1998) Microsatellite DNA markers reveal details of social structure in forest ants. Mol Ecol 7: 299-306.

Moilanen A, Sundström L, Pedersen JS (2003) MateSoft: a program for genetic analysis of mating systems 1.0b. University of Copenhagen, Copenhagen.

Palmer KA, Oldroyd BP, Quezada-Euán JJG, Paxton RJ, May-Itza WdJ (2002) Paternity frequency and maternity of males in some stingless bee species. Mol Ecol 11: 2107-2113.

Pamilo P (1993) Polyandry and allele frequency differences between the sexes in the ant *Formica aquilonia*. Heredity 70: 472-480.

Paxton RJ, Bego LR, Shah MM, Mateus S (2003) Low mating frequency of queens in the stingless bee *Scaptotrigona postica* and worker maternity of males. Behav Ecol Sociobiol 53: 174-181.

Paxton RJ, Thorén PA, Estoup A, Tengö J (2001) Queen-worker conflict over male production and the sex ratio in a facultatively polyandrous bumblebee, *Bombus hypnorum:* the consequences of nest usurpation. Mol Ecol 10: 2489-2498.

Sanetra M, Crozier RH (2001) Polyandry and colony genetic structure in the primitive ant *Nothomyrmecia macrops*. J Evol Biol 14: 368-378.

Strassmann JE, Nguyen JS, Arévalo E, Cervo R, Zacchi F, *et al.* (2003) Worker interest and male production in *Polistes gallicus*, a Mediterranean social wasp. J Evol Biol 16: 254-259.

Sundström L, Chapuisat M, Keller L (1996) Conditional manipulation of sex ratios by ant workers: a test of kin selection theory. Science 274: 993-995.

Tóth E, Strassmann JE, Nogueira-Neto P, Imperatriz-Fonseca VL, Queller DC (2002) Male production in stingless bees: variable outcomes of queen-worker conflict. Mol Ecol 11: 2661-2667.

Villesen P, Murakami T, Schultz TR, Boomsma JJ (2002) Identifying the transition between single and multiple mating of queens in fungus-growing ants. Proc R Soc Lond B 269: 1541-1548.

Walin L, Sundström L, Seppä P, Rosengren R (1998) Worker reproduction in ants - a genetic analysis. Heredity 81: 604-612.
